# The phosphodiesterase inhibitor, ibudilast, attenuates neuroinflammation in the MPTP model of Parkinson’s disease

**DOI:** 10.1371/journal.pone.0182019

**Published:** 2017-07-28

**Authors:** Joanna Schwenkgrub, Malgorzata Zaremba, Ilona Joniec-Maciejak, Agnieszka Cudna, Dagmara Mirowska-Guzel, Iwona Kurkowska-Jastrzębska

**Affiliations:** 1 Department of Experimental and Clinical Pharmacology, Centre for Preclinical Research and Technology (CePT), Medical University of Warsaw, Warsaw, Poland; 2 Laboratory of Magnetic Resonance Imaging of Small Animals, Mossakowski Medical Research Centre, PAS, Warsaw, Poland; 3 2^nd^ Department of Neurology, Institute of Psychiatry and Neurology, Warsaw, Poland; Rutgers University, UNITED STATES

## Abstract

**Background/Aims:**

Since the degeneration of the nigrostriatal dopaminergic pathway in Parkinson’s disease (PD) is associated with the inflammation process and decreased levels of cyclic nucleotides, inhibition of up-regulated cyclic nucleotide phosphodiesterases (PDEs) appears to be a promising therapeutic strategy. We used ibudilast (IBD), a non-selective PDE3,4,10,11 inhibitor, due to the abundant PDE 4 and 10 expression in the striatum. The present study for the first time examined the efficacy of IBD in the 1-methyl-4-phenyl-1,2,3,6-tetrahydropyridine (MPTP) mouse model of PD.

**Methods:**

IBD [0, 20, 30, 40, or 50 mg/kg] was injected b.i.d. subcutaneously for nine days to three-month-old male C57Bl/10Tar mice, beginning two days prior to MPTP (60 mg/kg) intoxication. High-pressure liquid chromatography, Western blot analysis, and real time RT-PCR methods were applied.

**Results:**

Our study demonstrated that chronic administration of IBD attenuated astroglial reactivity and increased glial cell-derived neurotrophic factor (GDNF) production in the striatum. Moreover, IBD reduced TNF-α, IL-6, and IL-1β expression.

**Conclusion:**

IBD had a well-defined effect on astroglial activation in the mouse model of PD; however, there was no protective effect in the acute phase of injury. Diminished inflammation and an increased level of GDNF may provide a better outcome in the later stages of neurodegeneration.

## Introduction

Parkinson’s disease (PD) is the second most common neurodegenerative disorder, affecting about 2% of the population over 65 years of age [1]. The currently available treatments improve some symptoms of the disease; however, they have sub-optimal efficacy that is related to the duration of the disease. A neuroprotective or disease modifying treatment is still needed [2, 3]. Since chronic neuroinflammation is associated with the pathogenesis of PD, the neuroinflammatory signaling pathways in the central nervous system (CNS) are of interest as potential pharmacotherapy targets [4]. Several candidate drugs directed at these targets have reached clinical trials but, unfortunately, none were effective. Experimental and clinical studies have demonstrated that the cyclic nucleotide phosphodiesterase (PDE) inhibitors may be useful in treating neuroinflammatory disorders.

There is increasing evidence in both experimental and clinical studies of PD to suggest that downregulation of the cyclic nucleotide mediated signaling cascade contributes to the striatal dysfunction [5–7]. Decreased levels of cyclic nucleotides lead to transcriptional dysregulation mediated by cAMP response element-binding protein (CREB) and, thus, decreased activity of neurotrophic factors, such as brain-derived neurotrophic factor (BDNF), nerve growth factor (NGF), neurotrophin-3 (NT-3), and neurotrophin-4 (NT-4) [6]. In addition, an increase in cytoplasmic cAMP concentration, resulting in the downregulation of nuclear factor kappa B (NF-κB), has been reported to reduce proinflammatory cytokines production and iNOS expression [8]. The level of cyclic nucleotides is controlled by PDEs that are grouped into 11 gene families with different substrate specificities, functions, regulation mechanisms, and tissue expression patterns. Members of the PDE1, PDE2, PDE4, and PDE10 families are highly expressed in the striatum [9–12]. It is postulated that inhibition of up-regulated PDEs may contribute to glial attenuation and be beneficial in movement and cognitive disorders [6, 13, 14]. The nigrostriatal-specific PDE10A, which is highly enriched in medium spiny neurons [10], may play a role in the pathophysiology of PD [15]. Inhibition of PDE10A results in a robust increase in cAMP and cGMP, as well as in elevation of phosphorylated CREB and BDNF in the striatum [16, 17]. Inhibition of PDE10A reduced striatal and cortical cell loss, the degree of microglial activation, and deficits in rotarod performance in a mouse model of Huntington’s disease [16]. It has also been reported that rolipram (a specific PDE4 inhibitor) exerts neuroprotective effects in the 1-methyl-4-phenyl-1,2,3,6-tetrahydropyridine (MPTP) model of PD, i.e. it attenuates MPTP-induced dopamine (DA) depletion in the striatum and protects against loss of dopaminergic neurons in the substantia nigra [18]. However, most of the selective inhibitors have failed during development due to intense gastrointestinal and cardiovascular adverse events [19–22]. According to the results of pre-clinical and clinical studies, a non-selective PDE3,4,10,11 inhibitor (ibudilast; IBD) is proposed as a better candidate for clinical evaluation than the same-class alternatives due to its efficacy and gastrointestinal tolerability [23]. In addition, some clinical data demonstrate a good safety record of IBD in both standard and higher-dose regimens [13]. Furthermore, we have a broad knowledge about the IBD safety due to its long-term presence on the market in Japan and other Asian countries, as a bronchodilator, vasodilator, and anti-inflammatory agent used for the treatment of asthma, post-stroke dizziness, and ocular allergies [13, 20, 24]. More recently, non-clinical data and clinical studies have indicated that IBD may have a broader range of action, clearly distinct from other well-studied PDE inhibitors, including suppression of pro-inflammatory cytokines (IL-1β, TNF-α, IL-6), leukotriene B4, nitric oxide production, allosteric inhibition of macrophage migration inhibitory factor (MIF) [24], and toll-like receptor 4 (TLR4) blockade. The documented spectrum of action also includes up-regulation of the anti-inflammatory cytokine (IL-10, IL-4) and promotion of neurotrophic factors (GDNF, NGF, NT-4) [13]. It is noteworthy that aside from its PDE inhibitory activity, IBD is inactive against various glial and/or neuronal targets [13, 25].

Studies demonstrated that IBD protects hippocampal neurons and oligodendrocytes against excitotoxicity, as well as astrocytes against apoptosis [26–28]. Furthermore, it has been shown to reduce white matter lesions induced by chronic cerebral ischemia [29]. In an animal model of experimental autoimmune encephalitis, IBD improved neurological function when administered prior to the onset of disease [30]. Since IBD is known to suppress glia cell activation (via undefined molecular mechanisms [13]), it is being evaluated for disease modifying effects in progressive multiple sclerosis and other neuroinflammatory conditions (e.g. neuropathic pain; medication overuse headache; and methamphetamine, alcohol, or opioid addiction; see www.clinicaltrials.gov for full listing). It is postulated that both glial attenuation and PDE inhibition contribute to the efficacy of IBD, and PDE inhibition alone may not be effective for neurological disorders [13]. The accumulated data suggest that IBD may be a promising therapeutic candidate for treating brain disorders due to its glial modulatory properties and good safety profile, activity on oral administration, and ability to cross the blood-brain barrier [20, 23]. These findings provide the rationale for examining the potential for IBD utility in PD. To the best of our knowledge, this will be the first study to assess the efficacy of the glia cell attenuator, a non-selective PDE inhibitor, and promoter of GDNF, in the animal models of PD.

The aim of this study was to evaluate the ability of investigated PDE inhibitor to prevent neuroinflammation and neurodegeneration in the MPTP model of PD.

## Materials and methods

### Animals

Mature adult (three months old, 27±3 g) male C57Bl/10Tar mice were used in the study. There were eight animals in each experimental group (except for 1-methyl-4-phenyl-tetrahydropyridinium ion [MPP^+^] evaluation, where we used four mice per group). All animals were housed in plastic breeding cages with free access to food and water, in controlled temperature (22±5°C) and 60±5% humidity, exposed to 12-h light/dark cycle. The experimental protocol was approved by the 2^nd^ Local Ethic Committee of the Medical University of Warsaw (Permit Number: 57/2013). Experiments were conducted according to the National Institutes of Health Guide for the Care and Use of Laboratory Animals, and in accordance with EU Directive 2010/63/EU. All efforts were made to minimize animal suffering and the number of animals needed to obtain reliable results.

### Experimental protocol

Mice were randomly divided into groups treated with either IBD (Shanghai Biochempartner Co., Ltd; China), or the vehicle. All of the solutions were prepared just before administration. The IBD [20, 30, 40, or 50 mg/kg] was dissolved in 35% poly(ethylene glycol) 400 (Sigma, USA) in saline and given b.i.d. for nine days with subcutaneous (s.c.) injections of 10 ml/kg body weight volume, beginning two days prior to MPTP intoxication. The MPTP group only received the MPTP intoxication.

#### Model induction

The PD was induced with MPTP-HCl (Sigma, USA) dissolved in saline (2 mg/ml), which was given to mice in four i.p. injections of 15 mg/kg each, at 1-hour intervals (cumulative dose: 60 mg/kg). The control group was injected with equivalent saline volume.

#### Preparation of tissue

Animals were sacrificed by spinal cord dislocation seven days post MPTP administration. The brain was rapidly removed, and both striata were dissected, weighed, and frozen at -80°C.

### High-pressure liquid chromatography (HPLC)

#### Evaluation of MPP^+^ content in the striatum

Assessment of the MPP^+^ level required additional experiments. Two hours prior to MPTP intoxication (injection of 40 mg/kg MPTP-HCl dissolved in saline [4 mg/ml]), IBD was administered to animals in doses similar to those described above. Mice were sacrificed two hours after MPTP treatment (four mice per group were used). Levels of MPP^+^ in dissected and immediately homogenized striata were analyzed by HPLC with a UV detector (wavelength: 295 nm). Twenty microliter aliquots were injected onto a reversed-phase column (125×3 mm with precolumn 5×3 mm; Nucleosil 120–3 C-18; Macherey-Nagel, Germany). The mobile phase was 0.14 M NaH_2_PO_4_ adjusted to pH 2.5 with H_3_PO_4_ in HPLC grade water containing 27,5% (v/v) acetonitrile, and was delivered at a flow rate of 0.5 ml/min. Data were analyzed in accordance with an external standard calibration.

#### Assay of dopamine and metabolites (3,4-dihydroxyphenylacetic acid, homovanillic acid)

The concentrations of DA and the corresponding metabolites (3,4-dihydroxyphenylacetic acid [DOPAC] and homovanillic acid [HVA]) were evaluated by HPLC with electrochemical detection (potential set at 0.8 V with respect to Ag/AgCl reference electrode) and a glassy carbon electrode. Samples were homogenized in ice-cold 0.1 M HClO_4_ solution and centrifuged (13,000 x g, 15 min) to precipitate proteins. The supernatant was filtered (0.2 μm pore size; Whatman, USA) and a 20 μl aliquot was injected onto the Nucleosil C-18 column, 250 mm, 5 μm particle size (Macherey–Nagel, Germany). The mobile phase was 32 mM sodium phosphate, 34 nM citric acid, 1 mM octanesulfonic acid, and 27 μM ethylenediaminetetraacetic acid (EDTA) (Sigma, USA) in deionized (18.3 mΩ) water and 12% methanol (Merck, Germany). The monoamines were separated using a 0.8 ml/min flow rate. Samples were quantified by comparing with standard (Sigma, USA) solutions (external calibration) using ClarityChrom software (Knauer, Germany).

### Immunoblotting

The tissue samples were homogenized with lysis buffer, centrifuged, and the supernatants were stored at -80°C. For the analysis of the target proteins, 65 μg of protein from each sample were separated on 15% SDS-polyacrylamide gels. Each gel contained lanes from the control and experimental groups. Proteins were electrophoretically transferred onto nitrocellulose membranes. Blots were incubated overnight in blocking solution (5% nonfat milk in TBST) containing the TH (rabbit polyclonal anti-TH, 1:1200, Millipore), GFAP (rabbit polyclonal anti-GFAP, 1:1200, Millipore), or Iba1 (goat polyclonal anti-Iba1, 1:200, Abcam) primary antibody. Detection was performed using an ECL Plus system (Amersham/GE Healthcare, Germany). The optical densities of bands were presented as their ratios to control lanes, determined with ImageJ software, version 1.48v (http://imagej.nih.gov/ij/). To avoid the risk of bias, the average of two control, as well as two MPTP lines were taken into account.

### Quantitative real time polymerase chain reaction (PCR)

To examine the TNF-α, IL-6, IL-1β, and GDNF changes at the transcriptional level, real time PCR analysis was performed using the DNA Engine Opticon 2 Real Time Fluorescence Detection System (MJ Research/Bio Rad). Total RNA was extracted from striatum using TRI reagent (Sigma, USA) and reverse transcribed with Moloney murine leukemia virus (Sigma, USA). The PCR primers used in this study were as follows: IL-1β (5′-AAA GAA GAA GAT GGA AAA GGC GGT T-3′; 5′-GGG AAC TGT GCA GAC TCA AAC TC-3′), IL–6 (5′-GAG GAT ACC ACT CCC AAC AGA CC-3′; 5′-AAG TGC ATC ATC GTT GTT CAT ACA-3′), TNF-α (5′-CAT CTT CTC AAA ATT CGA GTG ACA A-3′; 5′-TGG GAG TAG ACA AGG TAC AAC CC-3′), GDNF (5′-TGA CTC CAA TAT GCC TGA AGA TAT TC-3′; 5′-AAT GGT GGC TTG AAT AAA ATC CA-3′), and GAPDH (5′-TCT CCC TCA CAA TTT CCA TCC CAG-3′; 5′-GGG TGC AGC GAA CTT TAT TGA TGG-3′). The cDNA amplification was performed using the Maxima SYBR Green qPCR Master Mix (Thermo Scientific) and the following conditions: IL-1β: 50°C for 2 min, 95°C for 10 min, 40 x (95°C for 15 s, 60°C for 30 s); IL–6 and TNF-α: 50°C for 2 min, 95°C for 10 min, 40 x (95°C for 15 s, 60°C for 1 min, 72°C for 40 s); GDNF: 95°C for 10 min, 50 x (95°C for 15 s, 60°C for 1 min), 72°C for 1 min; and glyceraldehyde-3-phosphatedehydrogenase (GAPDH): 94°C for 15 min, 40 x (94°C for 30 s, 63.1°C for 30 s, 72°C for 30 s), 72°C for 5 min. The specificity of an amplified product was checked by melting curve analysis. Relative quantification was performed using Pfaffl’s method. The level of the housekeeping gene GAPDH was used as the reference standard to control amplification variations due to differences in mRNA quality and quantity. The mRNA expression of studied genes is presented as a relative unit determined by dividing the mRNA expression of the interest gene by the GAPDH gene.

### Statistical analysis

Data were statistically analyzed using STATISTICA 10 (StatSoft, Poland). All values are expressed as the mean ± SEM. The differences between groups of mice were analyzed by a nonparametric Mann-Whitney U-test; p < 0.05 was considered significant.

## Results

### MPP^+^ evaluation

The level of MPP^+^ after MPTP intoxication was measured to exclude pharmacological interactions between MPTP and IBD. Pretreatment with IBD [20, 30, 40, or 50 mg/kg] prior to MPTP intoxication did not affect the MPP^+^ concentration in the striatum (S1 Fig).

### Effect of ibudilast on the MPTP-induced nigrostriatal injury

To examine the potential neuroprotective effect of the studied compound on the injured striatal dopaminergic neurons in the MPTP-based model of PD, DA and its metabolites content in the striatum as well as TH protein expression were determined.

**Fig 1:**
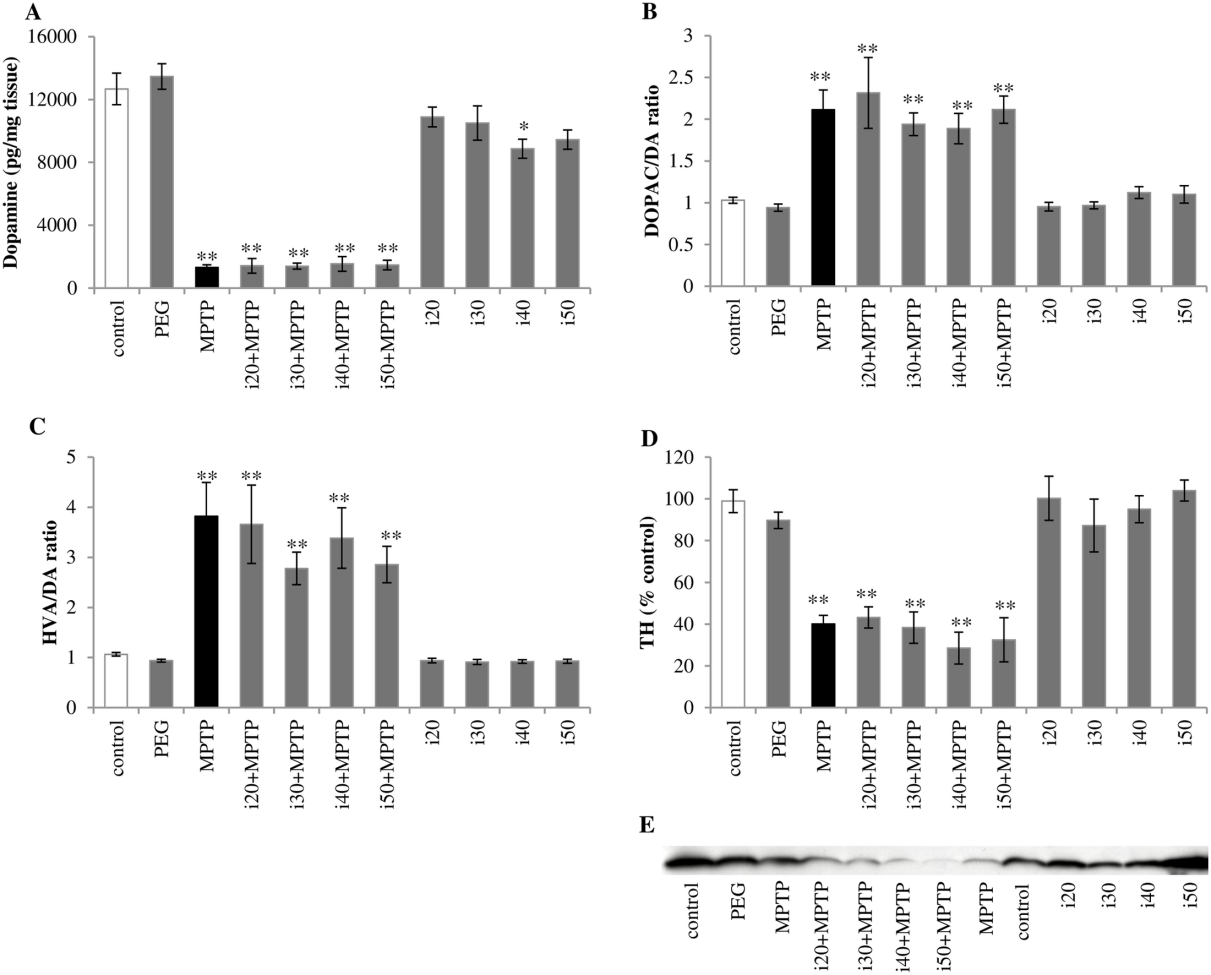
Effect of IBD on the injured nigrostriatal system seven days after MPTP intoxication. (A) DA concentration in the striata of C57Bl/10Tar mice, measured by HPLC. (B) The ratio of DOPAC to DA and (C) the ratio of final DA metabolite (HVA) to DA indicate the DA turnover rate. (D) Expression of tyrosine hydroxylase (TH) (the rate-limiting enzyme in catecholamine synthesis) in mice striatum was evaluated by Western blot analysis, and (E) one representative blot is shown of five independent experiments. Abbreviations of groups of animals: control–saline-treated; PEG–vehicle-treated; MPTP–MPTP-intoxicated; i(20, 30, 40, or 50)+MPTP–IBD [20, 30, 40, or 50 mg/kg] and MPTP-treated; i(20, 30, 40, or 50)–IBD [20, 30, 40, or 50 mg/kg] treated. The data are shown as means ± SEM (n = 5–6) (* represents a significant difference relative to the control group, *p<0.05, **p<0.01; Mann Whitney *U* test).

We investigated that MPTP treatment led to significant depletion of DA content, up to 10% of the control level in the striatum (Fig 1A) and an increase in DA turnover (DOPAC/DA and HVA/DA ratios) (Fig 1B and 1C). Concomitant treatment with IBD did not change the DA level and its turnover in MPTP-treated animals. However, 40 mg/kg IBD alone caused a decrease in DA concentration. The reduction in HVA/DA turnover was observed in mice treated with 40 or 50 mg/kg IBD alone. The TH protein expression represented 40% of the control level in MPTP-treated animals (Fig 1D), and IBD treatment did not affect the striatal TH expression.

### Effect of ibudilast on the inflammatory reaction in the striatum

To approve the anti-inflammatory effect of the studied compound in the MPTP-based model of PD, we investigated the changes in Iba1 (microglial marker) and GFAP (astrocytic marker) protein expression. We also evaluated the mRNA expression of pro-inflammatory cytokines (TNFα, IL-6, IL-1β) and a glial cell-derived neurotrophic factor (GDNF).

#### Effect of ibudilast on Iba1 and GFAP protein expression

**Fig 2:**
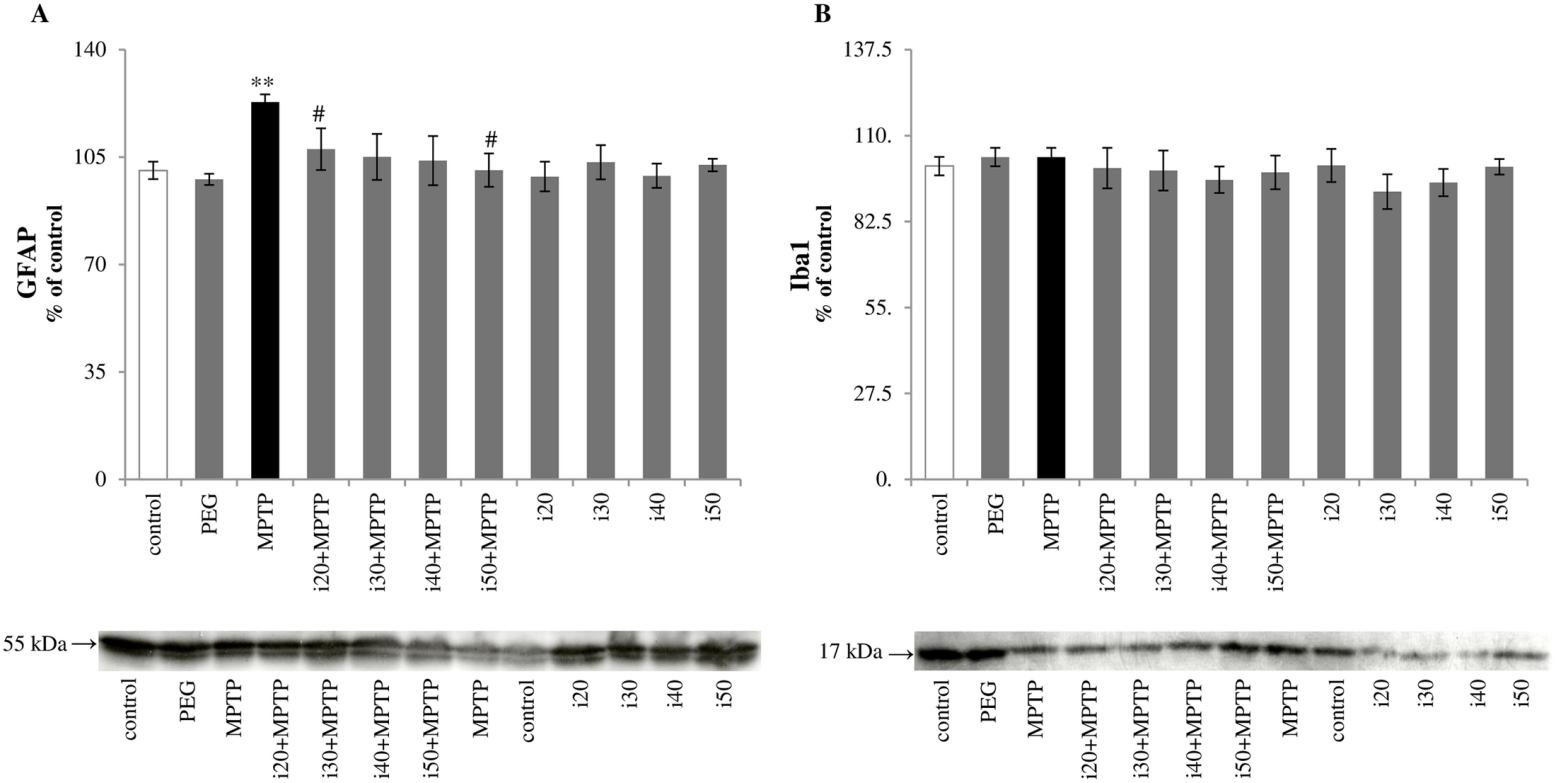
Protein expression of (A) GFAP and (B) Iba1 in the striatum, evaluated by immunoblot analysis. Abbreviations of groups of animals: control–saline-treated; PEG–vehicle-treated; MPTP–MPTP-intoxicated; i(20, 30, 40, or 50)+MPTP–IBD [20, 30, 40, or 50 mg/kg] and MPTP-treated; i(20, 30, 40 or 50)–IBD [20, 30, 40, or 50 mg/kg] treated. The values are expressed as mean ± SEM, for five mice per group (* represents a significant difference relative to control group, **p<0.01; ^#^ represents a significant difference compared with the MPTP group, ^#^p<0.05; Mann Whitney *U* test).

Western blot analysis showed no increase in Iba1 protein expression seven days after MPTP intoxication and IBD (Fig 2B) did not have an effect on the level of this microglial activation marker. The astrocytic reaction was assessed by analysis of GFAP protein expression. There was a tendency to suppress the MPTP-induced increase in GFAP expression in mice treated with PDE inhibitor. Statistically significant differences were found for animals treated with 20 or 50 mg/kg IBD (107.08±7.5 and 98.66±4.82 respectively vs. 122.93±2.52 in the MPTP group) (Fig 2A).

#### Effect of ibudilast on mRNA expression of pro-inflammatory cytokines

**Fig 3:**
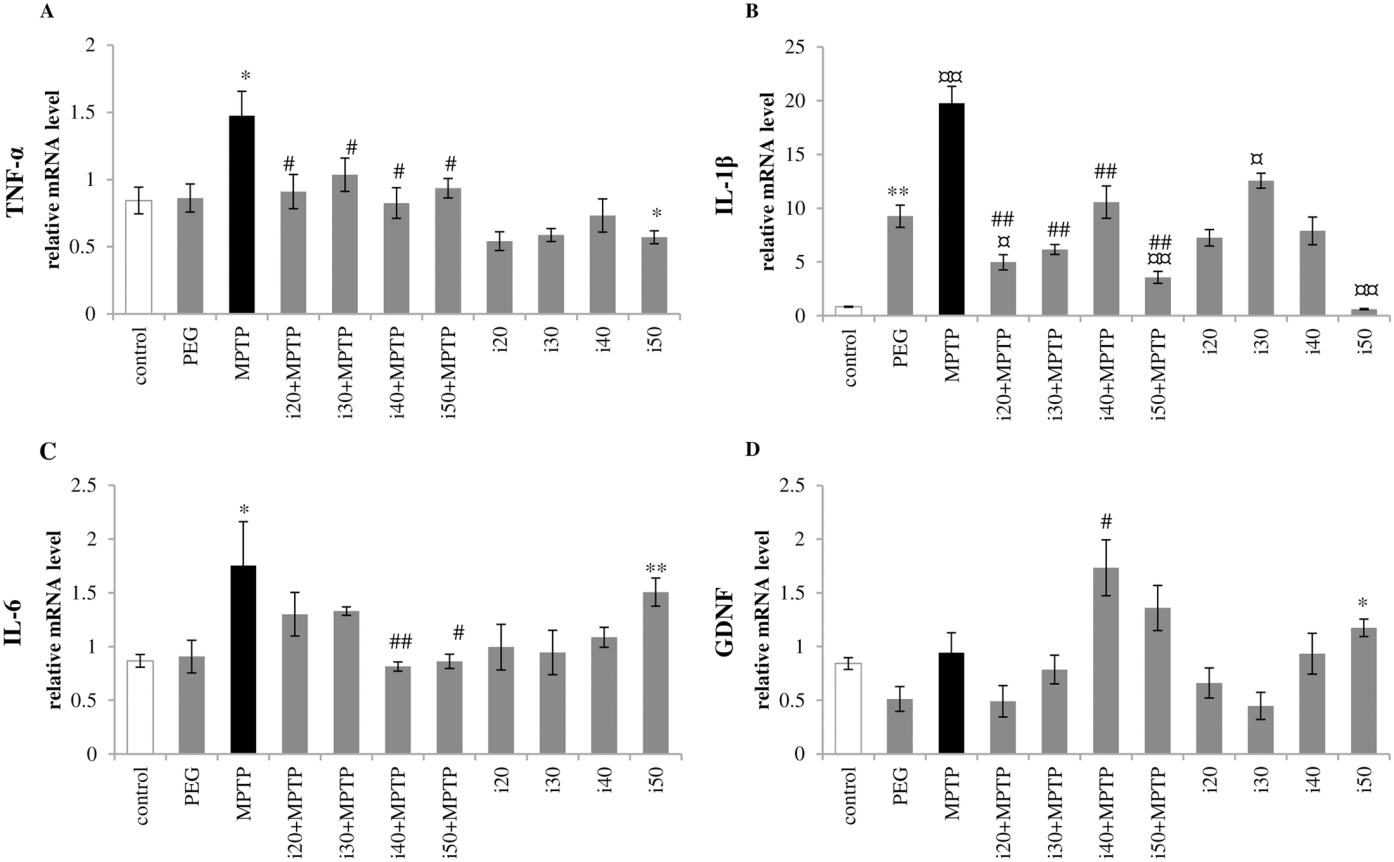
Effect of IBD on mRNA expression of pro-inflammatory cytokines and a trophic factor (GDNF). The mRNA expression of (A) TNF-α, (B) IL-1β, (C) IL-6, and (D) GDNF in the striatum was examined by real time RT-PCR. Abbreviations of groups of animals: control–saline-treated; PEG–vehicle-treated; MPTP–MPTP-intoxicated; i(20, 30, 40, or 50)+MPTP–IBD [20, 30, 40, or 50 mg/kg] and MPTP-treated; i(20, 30, 40, or 50)–IBD [20, 30, 40, or 50 mg/kg] treated. Data is given as the mean value ± SEM of five animals per group (* represents a significant difference relative to the control group, *p<0.05, **p<0.01; ^#^ represents a significant difference compared with the MPTP group, ^#^p<0.05, ^##^p<0.01; ^¤^ differs from vehicle-treated animals, ^¤^p<0.05, ^¤¤^p<0.01. Mann Whitney *U* test).

The real time RT-PCR analyses revealed significant reduction in mRNA expression of all investigated pro-inflammatory cytokines after IBD treatment. The IBD treatment can suppress the MPTP-induced increase in TNF-α (1.47±0.18 in the MPTP group vs. 0.84±0.10 in control) and IL-1β (19.76±1.58 in the MPTP group vs. 0.84±0.05 in control) for all investigated doses (Fig 3A and 3B). In addition, administration of 50 mg/kg IBD alone resulted in statistically significant decreases in TNF-α and IL-1β expression compared with the control level. The IL-1β mRNA expression was observed at a higher level than the control level (0.84±0.05) in animals treated with PEG (9.27±1.03) or 30 mg/kg IBD alone (12.55±0.69). The MPTP administration increased IL-6 expression in the striatum up to 201% of the control level (1.75±0.41 vs. 0.87±0.06 in control) (Fig 3C). This effect was attenuated by concomitant treatment with 40 or 50 mg/kg IBD (0.81±0.04 or 0.86±0.07, respectively). However, 50 mg/kg IBD alone increased expression of this pro-inflammatory cytokine.

#### Effect of ibudilast on mRNA expression of GDNF

The 40 mg/kg dose of IBD significantly increased production of the GDNF mRNA in mice intoxicated with MPTP (1.73±0.26 vs. 0.94±0.19 in the MPTP group) (Fig 3D); whereas, 50 mg/kg IBD alone increased the GDNF level in comparison with the control group (1.17±0.08 vs. 0.84±0.05).

## Discussion

We demonstrated that IBD treatment in the MPTP model of PD had no impact on striatal dopaminergic cell survival seven days after acute MPTP intoxication. However, the drug appeared to diminish glial cell activation. These results were not dependent on MPTP conversion to ion MPP^+^ (1-methyl-4-phenylpyridinium), which is responsible for inducing PD-like symptoms in mice, thus excluding simple interactions between MPTP and IBD [4]. In non-dopaminergic cells, MPTP is metabolized to 1-methyl-4-phenyl-2,3-dihydropyridinium (MPDP^+^) by monoamine oxidase B (MAO-B) and converted to MPP^+^ (probably by spontaneous oxidation). The toxic ion is subsequently released into the extracellular space and transported to dopamine neurons, where it inhibits mitochondrial complex I [31, 32].

It is known that MPTP injections cause damage to the nigrostriatal dopaminergic pathway, as seen in PD [33]. The dopaminergic neuron loss results in a drastic reduction in DA concentration and TH expression (the rate-limiting enzyme in catecholamine synthesis) [31]. Studies in the animal models of brain disorders demonstrated that treatment with PDE inhibitors might provide an efficient neuroprotective effect. Yoshioka et al. indicated a significant attenuation of cell density reduction in the hippocampal CA1 region in rats with transient cerebral ischemia treated with 10 mg/kg IBD [34]. It is also reported that pretreatment with IBD is effective in decreasing Aβ-induced apoptotic responses and cognitive impairment in mice [35]. In addition, the inhibition of PDE10A (the striatal-specific PDE) in a mouse model of Huntington’s disease resulted in significant amelioration in striatal cell survival, elevated levels of BDNF and phosphorylated CREB, as well as improvement in rotarod performance [16]. Nevertheless, in the present study, we did not find protective properties of IBD against neuronal death. The PDE inhibitor did not appear to have a relevant impact on the DA level or TH protein expression in the striatum (Fig 1A and 1D).

Negative result may suggest that IBD affects the PDE expression related to the pool of non-dopaminergic neurons in the striatum (e.g. GABAergic medium spiny neurons). Therefore, no significant change was observed in the expression of crucial enzyme for dopaminergic neurons.

Lesions of the nigrostriatal dopaminergic projections induce local inflammatory reaction with up-regulation of pro-inflammatory cytokines (such as IL-1β, IL-6, IL-8, and TNF-α) and the free radical release from activated glia [36–38]. In young mice, the microglial reaction starts on the 1^st^ day and is diminished on the 7th day following MPTP intoxication [39, 40]; while, the reactive astrogliosis is observed at least to 21 days post intoxication [41, 42]. Not surprisingly, no change in the expression of the microglial marker Iba1 was noted in this study (Fig 2B). However, immunoblot analysis revealed a significant increase in GFAP (a marker for astrocyte activation) at seven days following MPTP injections; although, reversal of this effect was observed in the groups additionally treated with IBD (Fig 2A). Furthermore, we noted that the changes in IL-6 expression (a mediator of astrogliosis [43]) corresponded with this phenomenon (Fig 3C).

It has been postulated that down-regulation of the inflammatory process in the MPTP-injured dopaminergic system may play a neuroprotective role [44]. Over-expression of anti-inflammatory cytokine IL-10 can restore the DA level and reduce the detrimental effects of MPTP intoxication in mice [45, 46]. Treatment with nonsteroidal anti-inflammatory agents (indomethacin or ibuprofen) led to similar results [38, 47]. In both *in vivo* models and *in vitro* studies, IBD inhibited microglia and astrocyte activation in a dose-dependent manner [8, 26, 34, 48]. The reduction in TNF-α, IL-1β, IL-6, and NO expression as well as enhanced production of IL-10 and neurotrophic factors (NT-4, GDNF, and NGF) are linked to its neuroprotective properties [8, 49]. In agreement with previous studies, we noted that the chronic treatment with IBD resulted in attenuation of MPTP-induced pro-inflammatory cytokine (TNF-α, IL-6, IL-1β) production in mice (Fig 3A–3C).

This study gives an overview of the neurotrophic potential of PDE inhibitor, which is manifested by a significant increase in GDNF level (Fig 3D). This effect was observed for MPTP-treated group of mice receiving higher dose of IBD (40 mg/kg). It is known that GDNF may prevent dopaminergic neurons from undergoing apoptosis [50]. Furthermore, GDNF treatment caused partial clinical recovery in parkinsonian monkeys [51, 52] as well as symptomatic improvement in human PD studies [53, 54]. However, the increase of GDNF in the present study did not protect dopaminergic neurons from MPTP-induced damage.

Our results obtained from this model need to be approved in the future by reproducible efficacy across multiple animal models to increase the chance for potential, successful translation. Overall, MPTP and other toxin-induced animal models of PD (6-OHDA, rotenone, paraquat, LPS) provided valuable insight into the cardinal motor symptoms of PD, related to dopaminergic deficits, as well as efficacy of classical antiparkinsonian drugs (e.g. levodopa, dopamine agonists, amantadine) [55–58]. In addition, anti-inflammatory compounds with potential neuroprotective capability for PD have been investigated using neurotoxin models in rodents [59]. Several of the above drugs efficiently work in the symptomatic treatment of PD. Although, we should still point out that most of the successful toxin-induced preclinical studies cannot be translated to clinical trials and new therapies, due to a lot of notable limitations existing at the preclinical and clinical stages [60]: first, there is incomplete understanding pathogenesis of PD related to interaction between the cell-autonomous and non-cell-autonomous pathways [60]; second, the lack of animal model that is a true pathocopy of PD, which mimics the global, progressive course of disease and alters multiple neural regions, networks as well as neurotransmitters [61]; third, deficiency in final proof of efficacy, obtained in non-human primate models, while complex cell death mechanisms may differ in rodents and primates [55]; fourth, heterogeneity of PD presentation with diverse response to medication in the clinical trials, thus monotherapy may not be sufficient to restore neuronal functions [62]; lastly, the lack of validated biomarkers for or the diagnosis and monitoring of treatment response; hence it is not possible to identify patients at the pre-motor stage of disease and to prove disease-modifying or the capabilities of neuroprotective agents at the optimal intervention time [60, 62].

## Conclusions

The PDE inhibitor, IBD, acts as anti-inflammatory agents in the MPTP model of PD. Given the results of the present study, we hypothesize that IBD may exert its therapeutic effect over time via modulating glial cell activity. In addition, the attenuation of TNF-α expression and induction of GDNF in the acute phase of neurodegeneration did not exert a protective effect. Further studies will be focused on whether PDE inhibitor shows different restorative potential in treated and untreated animals over longer observation times.

## Supporting information

S1 Fig MPP^+^ evaluation. The amount of MPP^+^ in the striatum, measured two hours after MPTP administration to mice treated with 35% PEG in saline (vehicle) or various doses of IBD. Abbreviations of groups of animals: MPTP–treated with 40 mg/kg MPTP; PEG+MPTP–treated with vehicle prior to MPTP intoxication; i(20, 30, 40, or 50)+MPTP–IBD-treated [20, 30, 40, or 50 mg/kg] prior to MPTP injection. The data are shown as means ± SEM (n = 4) (Mann Whitney U test).
(TIF)
