# Supplementary figures and images for: The phosphodiesterase inhibitor, ibudilast, attenuates neuroinflammation in the MPTP model of Parkinson’s disease
Source: PLoS One. 2017 Jul 28;12(7):e0182019. doi: 10.1371/journal.pone.0182019 (PMC5533435; doi:10.1371/journal.pone.0182019)

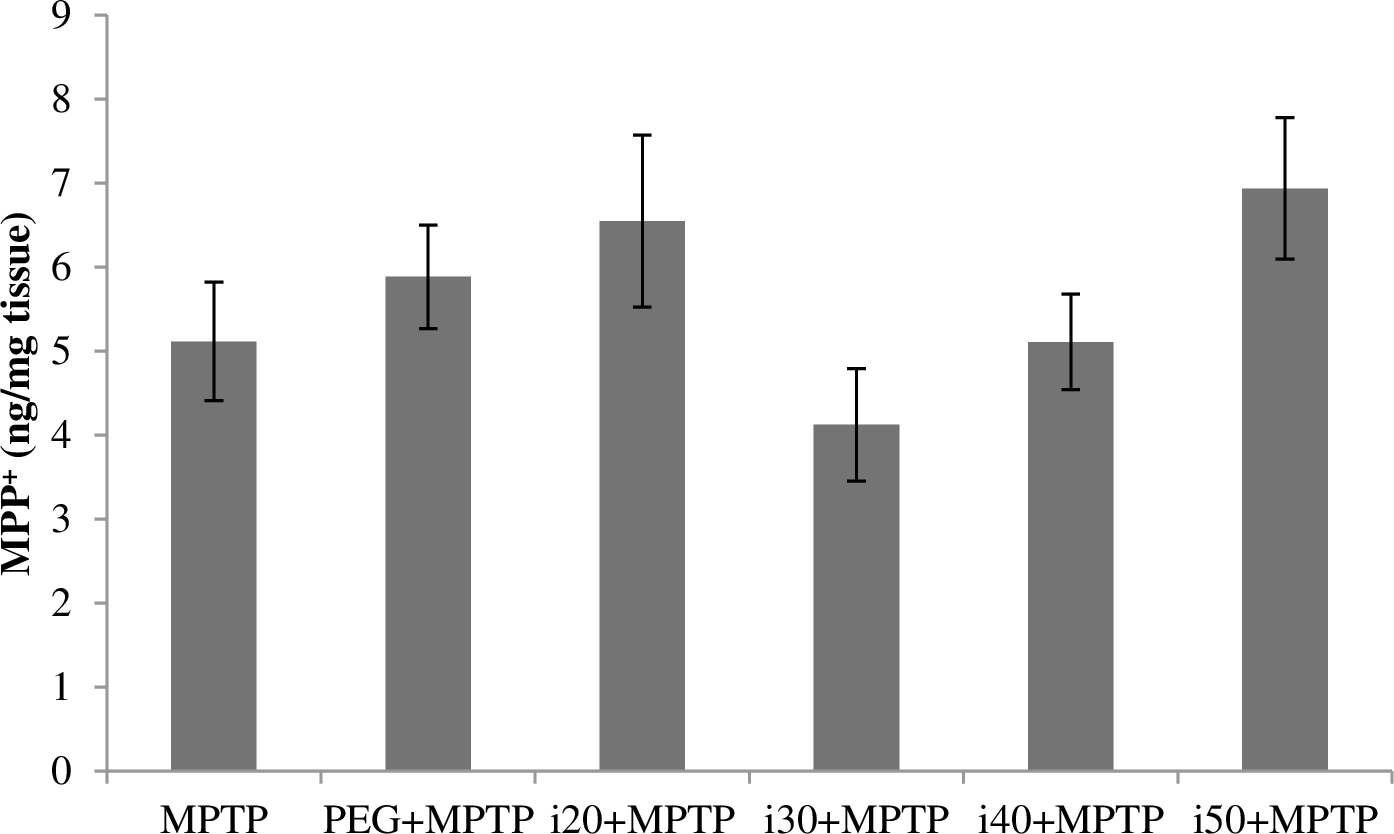

Supplement: S1 Fig — The amount of MPP+ in the striatum, measured two hours after MPTP administration to mice treated with 35% PEG in saline (vehicle) or various doses of IBD. Abbreviations of groups of animals: MPTP–treated with 40 mg/kg MPTP; PEG+MPTP–treated with vehicle prior to MPTP intoxication; i(20, 30, 40, or 50)+MPTP–IBD-treated [20, 30, 40, or 50 mg/kg] prior to MPTP injection. The data are shown as means ± SEM (n = 4) (Mann Whitney U test). (TIF) [file pone.0182019.s001.tif]
